# Pyoderma Gangrenosum Masquerading as Wound Infection in the Early Postoperative Period After Lumbar Spine Deformity Correction Surgery

**DOI:** 10.7759/cureus.25545

**Published:** 2022-05-31

**Authors:** Bryce S Owen, Mark A Pacult, Bryan S Lee

**Affiliations:** 1 Department of Neurosurgery, St. Joseph’s Hospital and Medical Center, Barrow Neurological Institute, Phoenix, USA

**Keywords:** surgical site infection, spinal fusion, spine surgery, pyoderma gangrenosum, postoperative infection

## Abstract

The development of pyoderma gangrenosum (PG) after surgical site trauma is a rare, poorly understood immunologic phenomenon. PG is an immunologic disorder characterized by lymphocytic infiltration of the dermis that can manifest with skin necrosis and ulceration. This rare phenomenon can mimic surgical site infection (SSI) when it occurs in the perioperative period and in the region of surgical wounds. Within the neurosurgical literature, only two cases of postoperative PG have been reported to our knowledge. We describe the clinical features and treatment of PG in the case of a 65-year-old man who underwent a three-stage surgical approach for intractable mechanical low back pain on hospital days (HDs) 1 and 2, and who subsequently developed PG around all three surgical sites in the immediate postoperative period (HD 8). The physical and laboratory findings and surgical and pharmacologic treatments are detailed. The patient was initially treated for presumed SSI, started on broad-spectrum antibiotics, and underwent surgical wound debridement twice, without resolution of symptoms. The diagnosis of PG was ultimately made by a consulting dermatologist on HD 17. The patient was started on systemic immunosuppression with steroids during his initial hospitalization; symptoms resolved within two weeks of the index surgery. Although PG is a rare entity, we suggest that it be considered in the differential diagnosis of nonhealing surgical wounds. Familiarity with PG may help mitigate unnecessary surgical morbidity and reduce the length of hospital stays and unnecessary use of antibiotics.

## Introduction

Pyoderma gangrenosum (PG) is a rare immunologic phenomenon that mimics a surgical site infection (SSI). PG typically manifests as painful skin pustules that can rapidly progress to large necrotic ulcers with raised violaceous borders [1]. Because skin conditions caused by PG may appear to be necrotic, PG is often misdiagnosed as an SSI, which can lead to delays in definitive treatment, unnecessary procedures, and significantly increased healthcare costs [2]. The pathogenesis of PG, which occurs in tandem with other autoinflammatory diseases, is thought to be due to the dysfunctional activation of neutrophils and aberrant T cell activation and cytokine stimulation [3-5]. This hypersensitivity reaction may be triggered by skin trauma, which is a phenomenon known as pathergy, and it occurs in less than 1% of patients with PG [6-8]. Postoperative PG is most commonly described after surgery involving the abdomen or breast [9-14]. Only three studies note PG appearing in the postoperative setting after spine surgery [9,15,16].

This study details the case of postoperative wound dehiscence after spine surgery that was initially treated as an SSI with two debridements and washouts; these treatments ultimately did not result in improvement of the wound. A diagnosis of PG was made nearly two weeks after index surgery, and the patient immediately improved after empiric treatment for PG. Explicit patient consent was obtained to disclose the health information in this study. Institutional review board review was not sought because a single case report does not meet the minimum criteria for institutional review board review.

## Case presentation

**Figure 1:**
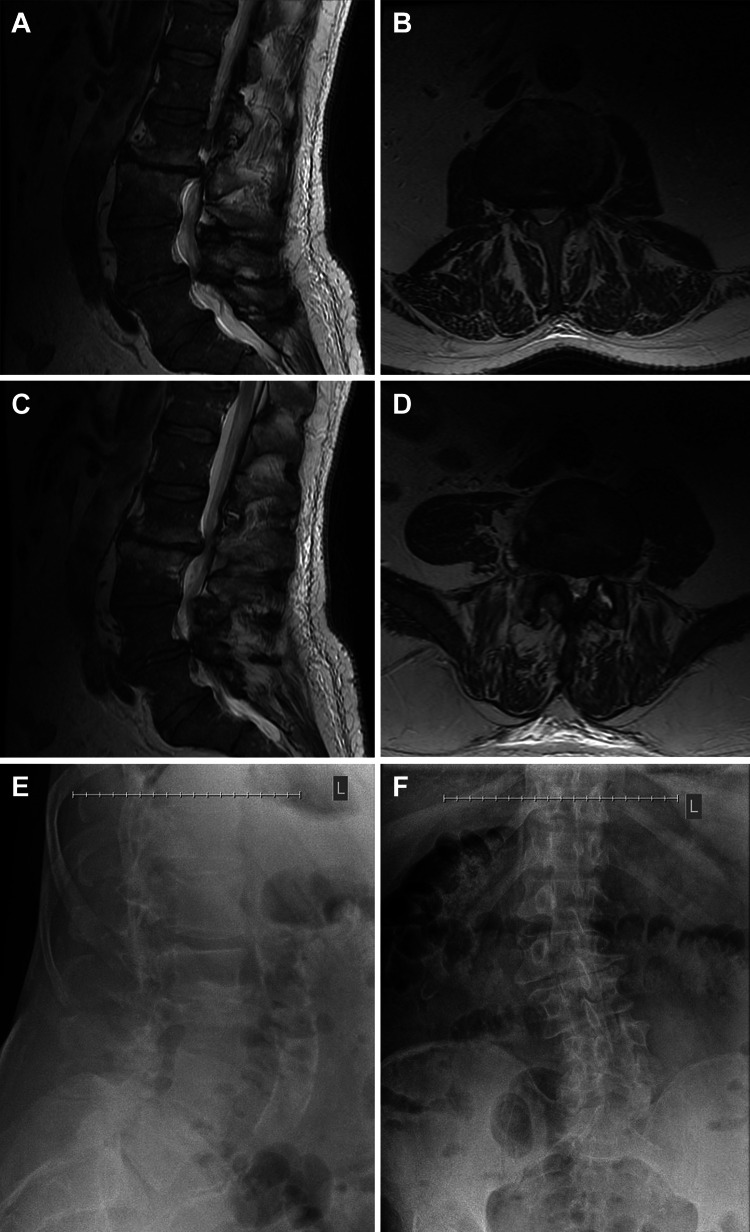
Preoperative lumbar imaging of the patient. Magnetic resonance images showing severe central and foraminal stenosis at L2-3 on (A) sagittal and (B) axial views, and at L4-5 on (C) sagittal and (D) axial views. (E) Lateral and (F) anteroposterior radiographs demonstrating dextroscoliosis, grade 1 L2-3 retrolisthesis, and grade 1 L4-5 anterolisthesis. The image is used with permission from Barrow Neurological Institute, Phoenix, Arizona.

A 65-year-old man without significant medical history or history of prior surgery presented after conservative management failed to alleviate his chronic mechanical back pain, left leg radiculopathy, and paresthesia. Preoperative imaging obtained at presentation demonstrated levoscoliosis of the lumbar spine with spondylolisthesis, severe stenosis, and advanced degenerative findings at multiple lumbar segments (Figures 1A-1F). The patient was neurologically intact without any focal deficits.

**Figure 2:**
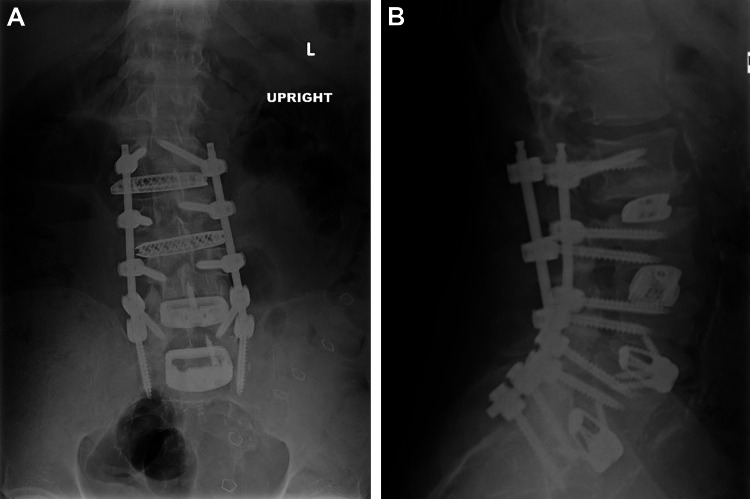
Postoperative (A) posterior and (B) lateral lumbar radiographs. Radiographs demonstrate the placement of extreme lateral interbody fusion cages in the L2-3 and L3-4 disk spaces, as well as anterior lumbar interbody fusion cages at L4-5 and L5-S1, with bilateral screws and rods placed percutaneously from L2 to S1. The image is used with permission from Barrow Neurological Institute, Phoenix, Arizona.

A three-stage surgical approach was recommended with L4-5 and L5-S1 anterior lumbar interbody fusion, L2-3 and L3-4 extreme lateral interbody fusion, and posterior L2-S1 percutaneous screw placement and arthrodesis to correct the patient’s degenerative spondylolisthesis and to indirectly decompress the neural foramina. The patient underwent stage 1 and 2 procedures on hospital day (HD) 1 and stage 3 on HD 2 without any acute complications and with satisfactory postoperative images (Figures 2A, 2B). A timeline of the patient’s hospital course is detailed in Table 1.

**Table 1:** A timeline of the hospital course from surgery to discharge of a patient with pyoderma gangrenosum masquerading as a wound infection. ALIF: anterior lumbar interbody fusion; OR: operating room; XLIF: extreme lateral interbody fusion

Hospital Day	Treatments, Outcomes, and Complications
1	Stage 1 L4-S1 ALIF and L2-4 XLIF
2	Stage 2 posterior L2-S1 percutaneous screw placement; afebrile
3-4	No acute complications; standard perioperative antibiotics administered, but no steroids given; satisfactory postoperative imaging
5	White blood cell count at 12.0 x 10^9 ^cells/L
6-7	Wound begins to dehisce and express milky fluid.
8	Abdominal wound continues with dehiscence and ulceration; patient taken to OR for wound washout; culture negative
9-11	Wound cultures remain negative; white cell count increases to 19.8 x 10^9^ cells/L
12	Wound unimproved; patient taken to OR for second wound washout and debridement
13	Wound ulceration progresses
14-16	Wound continues to ulcerate and dehisce despite antibiotic therapy
17	Dermatologic consultation obtained; intravenous steroids initiated to treat clinical diagnosis of pyoderma gangrenosum
18	Improvement in wound ulceration and edema; patient discharged home

**Figure 3:**
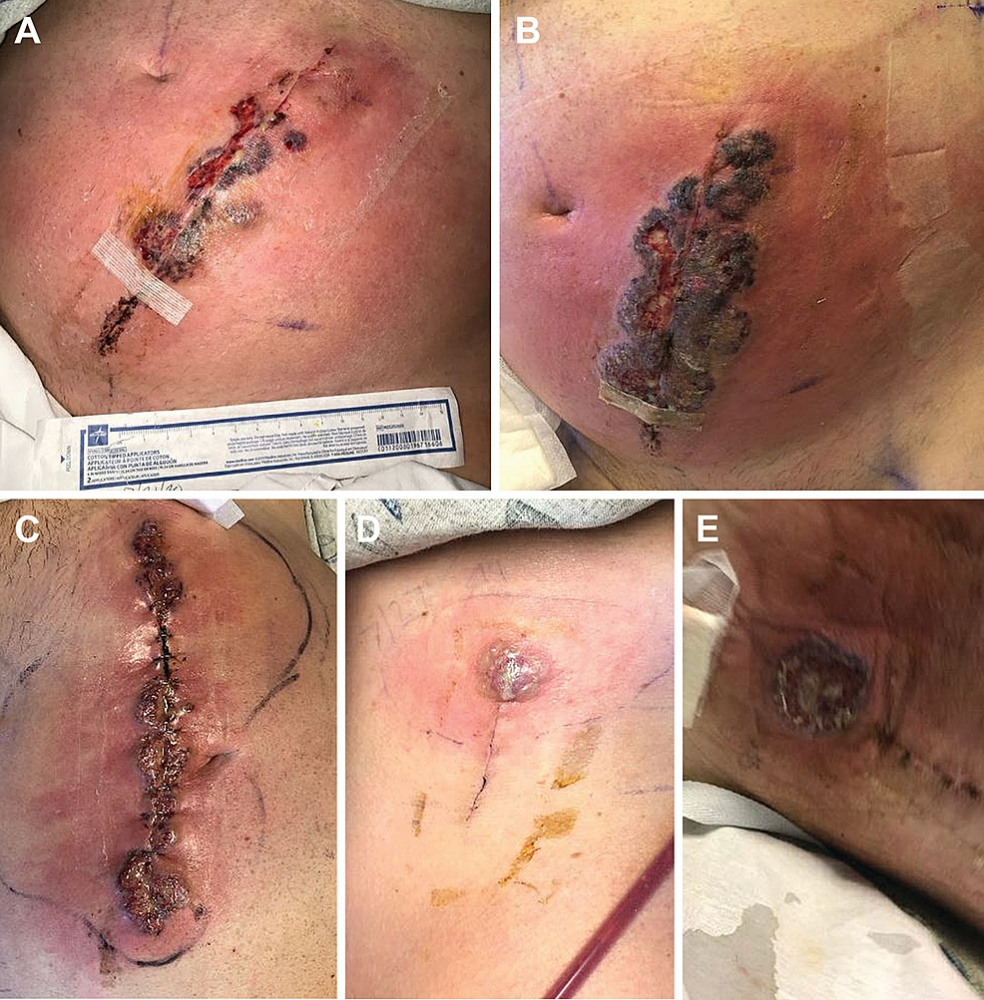
Postoperative wound photographs. (A) Abdominal wound on hospital day (HD) 8. (B) Abdominal wound on HD 9 shows increased swelling, redness, and growing region of ulceration. (C) Abdominal wound several days after first excisional debridement and washout with recurrent purulent drainage, redness, and raised bluish wound borders. (D) Lateral surgical wound on HD 13 shows similar features of redness, swelling, and ulceration. (E) Posterior surgical site on HD 13 with a large area of central necrosis and violaceous wound border. The image is used with permission from Barrow Neurological Institute, Phoenix, Arizona.

The patient had an unremarkable postoperative course in the initial four days; he received standard perioperative antibiotics in the form of intravenous cefazolin (2g every eight hours) but not steroids. On HD 5, the patient’s white blood cell count was elevated at 12.0 x 10^9^ cells/L, although he was afebrile. On HD 8, the patient’s anterior wound began to express purulent fluid, with the wound edges showing dehiscence and ulceration (Figure 3A). Initial intravenous cefazolin therapy (2g every eight hours) was broadened to linezolid and piperacillin/tazobactam. The anterior wound continued to ulcerate, and the surrounding abdominal area became erythematous and edematous (Figures 3B, 3C). The decision was made to proceed with an abdominal wound washout under the presumption that the wound was infected. All surgical wound cultures remained negative at 48 hours. However, the white blood cell count continued to increase, and the wound remained erythematous and purulent. On HD 12, the wound remained unimproved, and the patient again underwent another excisional debridement and washout. On HD 13, the posterior and lateral wounds appeared erythematous, swollen, and ulcerated (Figures 3D, 3E). Dermatology consultation was ultimately obtained on HD 17, with recommendations for steroid initiation for possible PG, which was diagnosed clinically. Subsequently, it was revealed by his family that he has a significant family history of inflammatory bowel disease; family members with the disease managed their condition with chronic use of immunotherapy.

**Figure 4:**
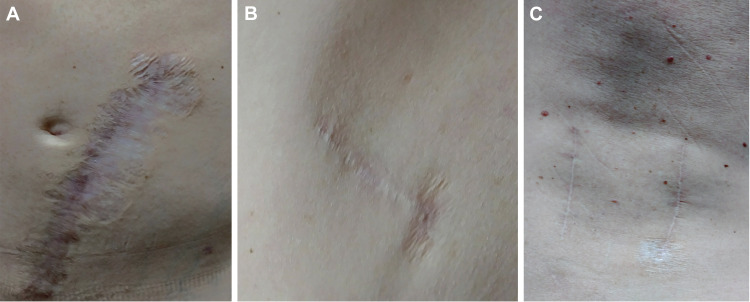
Wound photographs taken at the most recent one-year follow-up visit. (A) Abdominal wound corresponding to Figure 3A. (B) Lateral wound corresponding to Figure 3D. (C) Posterior wound corresponding to Figure 3E. The image is used with permission from Barrow Neurological Institute, Phoenix, Arizona.

After initiating intravenous prednisolone at a dosage of 50 mg, the patient’s abdominal wound improved within several hours with a noticeable decrease in erythema and edema. The patient was discharged home on HD 18 on oral amoxicillin/clavulanate (875/125 mg twice daily), oral doxycycline (100 mg twice daily), and a tapering dose of steroids starting at 50 mg for the first of seven days. At the six-week postoperative follow-up, the patient showed significant improvement in preoperative back pain, and his wounds were completely healed (Figures 4A-4C).

## Discussion

This case illustrates the development of a nonhealing wound in the immediate postoperative period after spine surgery. Only three cases of postoperative PG after spine surgery have been reported, with only one of these reported in the neurosurgical literature [9,15,16]. The first case, reported more than 20 years ago in the orthopedic literature, describes a 42-year-old man who underwent an open anteroposterior vertebral arthrodesis and developed dehiscence of his abdominal wound on HD 4 [15]. The patient was treated for a presumed SSI, but his condition continued to deteriorate until complete abdominal evisceration occurred on HD 9. At this point, a dermatologist was consulted and recommended systemic immunosuppression for presumed severe PG. The second case, which is the only case reported in the neurosurgical literature, describes a 71-year-old woman who developed PG one month after undergoing T4-ilium re-instrumentation [9]. This patient was managed conservatively for weeks with local wound care and antibiotic therapy before a referral to dermatology was obtained and treatment with steroid therapy resulted in improvement of wound appearance. The third case describes a patient with PG, confirmed histopathologically, that began at the surgical site nearly four weeks after surgery [16]. The patient’s wound was treated as a presumed SSI with initial drainage and debridement. Histopathological diagnosis was obtained from a distal IV site after the failure of improvement. In all of these reported cases, the ulceration was presumed to be caused by an SSI, and the patients were started on antibiotic treatment. However, the diagnosis of PG was delayed. Our case is the first reported case of immediate manifestation in the perioperative, inpatient setting. Our case demonstrates the rare involvement of all three surgical sites, with immediate resolution of symptoms after initiating systemic steroid treatment. Despite the rarity of PG, our report demonstrates that postoperative PG should be considered in patients with surgical wounds that appear infected in the immediate postoperative period (HDs 1-7) when SSIs are less likely to manifest.

When patients present with possible SSIs in the immediate postoperative period, the possibility of PG should be considered, especially in patients with a medical or family history of systemic inflammatory disease, such as inflammatory bowel disease, although the majority of cases develop without such history [2,7]. PG lesions typically erupt between four days to six weeks postoperatively. These lesions are often painful with a surrounding zone of redness that ulcerates and necrotizes as it expands, and the patient’s inflammatory markers and temperature are usually elevated [17]. Treatment involves immunosuppression, usually in the form of steroids or cyclosporine [18-20]. Response to immunosuppression is typically seen within days of treatment initiation. However, it may take weeks to months for the ulceration to completely heal. Thus, a steroid taper is usually initiated once clinical improvement manifests to avoid adverse effects of long-term steroid use [20]. All patients should be monitored for signs of reoccurrence.

It is not uncommon for patients ultimately diagnosed with PG to first undergo treatment for presumed SSI [2,17]. However, in patients without clinical improvement after appropriate antibiotic treatment and surgical reoperation for SSI, the suspicion for an alternative diagnosis must be raised. A timely diagnosis of PG is critical because additional skin trauma from surgical reoperation worsens the clinical course due to pathergy.

## Conclusions

Although postoperative PG is exceedingly rare, our case suggests that familiarity with this entity may mitigate costly and untimely surgical morbidity, the length of hospital stays, and the unnecessary use of antibiotics. Continuous wound ulceration, purulence, and erythema surrounding the surgical site in the immediate postoperative period despite antibiotic therapy and surgical reoperation, as well as a medical or family history of systemic inflammatory disease, should raise the suspicion of a noninfectious etiology. Prompt dermatologic consultation and treatment with immunosuppression should be pursued in such cases. Biopsy may prove useful at the time of wound washout to obtain a histologic diagnosis, though this was not pursued in our case. Ultimately, a short tapering course of steroids to treat PG should not have implications for bony fusion in patients who have undergone spine surgery and treatment for PG should be safe to pursue.
